# Expansion or compression of long-term care in Germany between 2001 and 2009? A small-area decomposition study based on administrative health data

**DOI:** 10.1186/s12963-016-0093-1

**Published:** 2016-07-13

**Authors:** Daniel Kreft, Gabriele Doblhammer

**Affiliations:** Institute for Sociology and Demography, University of Rostock and Rostock Center for the Study of Demographic Change, Ulmenstraße 69, D-18055 Rostock, Germany; Institute for Sociology and Demography, University of Rostock, German Center for Neurodegenerative Diseases, and Rostock Center for the Study of Demographic Change, Ulmenstraße 69, D-18055 Rostock, Germany

**Keywords:** Expansion, Compression, Dynamic equilibrium, Healthy life expectancy, Regions, Care need, Mortality, Regression, Time trends, Health inequality

## Abstract

**Background:**

Studies state profound cross-country differences in healthy life years and its time trends, suggesting either the health scenario of expansion or compression of morbidity. A much-discussed question in public health research is whether the health scenarios are heterogeneous or homogeneous on the subnational level as well. Furthermore, the question arises whether the morbidity trends or the mortality trends are the decisive drivers of the care need-free life years (CFLY), the life years with care need (CLY), and, ultimately, the health scenarios.

**Methods:**

This study uses administrative census data of all beneficiaries in Germany from the Statutory Long-Term Care Insurance 2001–2009. We compute the CFLY and CLY at age 65+ for 412 counties. The CFLY and CLY gains are decomposed into the effects of survival and of the prevalence of care need, and we investigate their linkages with the health scenarios by applying multinomial regression models.

**Results:**

We show an overall increase in CFLY, which is higher for men than for women and higher for severe than for any care need. However, spatial variation in CFLY and in CLY has increased. In terms of the health scenarios, a majority of counties show an expansion of any care need but a compression of severe care need. There is high spatial heterogeneity, with expansion-counties surrounding compression-counties and vice versa, which is mainly caused by divergent trends in the prevalence of care need. We show that mortality is responsible for the absolute changes in CFLY and CLY, while morbidity is the decisive driver that determines the health scenario of a county.

**Conclusion:**

Combining regionalized administrative data and advanced statistical methods permits a deeper insight into the complex relationship between health and mortality. Our findings demonstrate a compression of life years with severe care need, which however, depends on the region of residence. To attenuate regional inequalities, more efforts are needed that improve health by medical and infrastructural interventions and by the exchange of insights in the efficiency of small- and large-area policy measures between the vanguard and the rearguard counties. In future research, the underlying latent mechanisms should be investigated in more detail.

**Electronic supplementary material:**

The online version of this article (doi:10.1186/s12963-016-0093-1) contains supplementary material, which is available to authorized users.

## Introduction & Background

### The health scenarios

Three hypothetical scenarios with contrasting assumptions about future developments of morbidity in populations with decreasing mortality were established and repeatedly examined. In Ernest Gruenberg’s [1] and Morton Kramer’s [2] theory of “Expansion of Morbidity,” the general survival progress and the later-active (or even missing) improvements in health prevention and in recovery lead to an increasing duration of morbidity and a higher prevalence of health limitations. The contrary hypothesis is the “Compression of Morbidity” scenario by James Fries [3, 4], in which a general decrease in the incidence of morbidity is expected due to, for example, a healthier life style of the individuals, technological and medical advancements, and interventions in primary and secondary prevention of diseases. The morbidity shrinkage – combined with steadily improved survival rates – causes a postponement of unhealthy life years into the very last ages of life and results in a decline of the population’s prevalence of chronic diseases in total. Fries [5, 6] later developed a modified and differentiated scenario: the absolute and the relative compression of morbidity. Absolute compression describes a situation in which the total number of unhealthy life years decreases, while there is a relative compression when the proportion of unhealthy life time to total remaining life time declines. Furthermore, relative compression is defined as a special case of absolute compression - differing in the development of the disabled life years. If the number of disabled life years is stable or shrinking, there is an absolute compression, and if there is a slight increase in the number of disabled life years (but lesser than the gain in non-disabled life years), then the situation is defined as a relative compression.

In conjunction with the compression scenarios, there are two expansion scenarios: the absolute and the relative expansion of morbidity. The total number of unhealthy life years increases in the absolute expansion scenario, while the proportion of unhealthy life time to total remaining life time gains in the relative compression scenario.

The idea of looking at relative more than at absolute changes in morbidity prevalence rates is one of the basics of the theory of “dynamic equilibrium” [7, 8]. This scenario integrates the frameworks of compression and expansion of morbidity. Manton [7] assumes that gains in life expectancy go together with increasing years in ill-health; however, the share of unhealthy to total remaining life years remains relatively constant. Furthermore, while the total number of persons with chronic diseases is growing, the prognosis according to the theory expects a shift from more to less and moderate severe diseases and disability states. Behavioral, technological, and medical progression are the causes of this redistribution and will lead to a general improvement in survival as well [9].

To evaluate these frameworks of the future trends in population’s health status, summary measures were developed that combine information about morbidity and mortality data. One appropriate concept is the above-mentioned care need-free life years (CFLY). By combing the CFLY with the indicators life years with care need (CLY) and the health ratio (HR), five theoretical health scenarios can be identified (Table 1).

**Table 1:** Scheme of combinations of care need-free life years, life years with care need and health ratio by scenario of future health development (given that life expectancy increase continues)

	Disability-free life years	Disabled life years	Health ratio
Absolute Compression	▲	▼=	▲
Relative Compression	▲	▲	▲
Dynamic Equilibrium^a^	▲	▲	=
Relative Expansion	▲	▲	▼
Absolute Expansion	▼=	▲	▼

*Note*: ▼: decrease; ▲: increase; =: stable

^a^With considering the shift in the severity of morbidity, special case of “stability” that is defined by the same scenario but without considering the shift in the severity of care need

Until now, the CFLY indicator has been predominantly used for cross-country comparisons of time trends (e.g., [10, 11]). However – as mentioned above – the CFLY can equally be applied for regional comparisons within a country (e.g., [12, 13]).

A methodological problem occurs when time trends are studied based on changes in prevalence, because they can be the result of changes in the incidence, in the mortality of the prevalent population, and in the mortality of the non-prevalent population. Because longitudinal data on these three influencing factors are rare, Nusselder and Looman [14] introduced a decomposition method that allows for the retrospective separation of changes in prevalence (morbidity effect), changes in the survival of the population with morbidity (mortality effect on CLY, Mort_ΔCLY_), and changes in the survival of the population without morbidity (mortality effect on CFLY, Mort_ΔCFLY_). The two morbidity effects on CFLY and on CLY, are – by definition of a two-state decrement life table – the same in numbers but with opposite signs. A positive morbidity effect is defined as a decrease in prevalence. A positive Mort_ΔCFLY_ implies a decrease of mortality rates in the population without care need, and a positive Mort_ΔCLY_ is a decrease of mortality in the population with care need.

### Factors of and time trends in care need

#### Factors of care need

In our study care need is a complex, multidimensional concept of morbidity in contrast to the widely used health outcomes such as limitations in (instrumental) activities of daily living (ADL and IADL), self-rated health, or mobility limitations. Our measure of care need is based not only on an objective medical assessment of health problems due to IADL and ADL limitations according to the German Statutory Long-Term Care (SLTC) insurance, but also on the willingness to apply for benefits (see data section below).

In general, the risk of care need is affected by various determinants that can be differentiated into micro- and macro-level factors. Kibele [15], by adapting [16, 17], defines four subgroups of micro-level determinants: the socioeconomic status, lifestyle, living conditions, and human biology/genetic factors. Even if these factors are situated on the individual level, there is a spatial variation in the concentration of persons with specific health promoting or jeopardizing attributes in Germany (e.g., see review by [15]). In addition, three macro-level determinants can be identified: socioeconomic conditions, medical care provision, and environmental conditions (e.g., see review by [15]). Both micro- and macro-level factors influence – in a complex, interfering, and interplaying way - the disease burden situation in the particular German counties. Profound county differences were reported for a series of diseases and conditions all related to care need, such as dementia and hearing impairments [18], multiple sclerosis [19], smoking and obesity [20], depression [21], hypertension, diabetes mellitus, hypercholesterolemia [22], and acute stroke admission [23]. County differences also exist for all leading causes of death [15].

In addition, the decision to apply for SLTC- benefits depends on the individual and his or her family. In 2013, 71 % of the SLTC-beneficiaries received outpatient and informal care in their private homes; among these 66 % were only cared for by a family member [24]. Thus, the family (primarily the spouse and/or the children) plays an important role as care givers in the SLTC-system. One may assume that in most cases the decision of whether to apply for SLTC-benefits is discussed with and supported by the partner and the family. Potential factors influencing this decision may be the availability of a (distant) living partner/children, the amount of financial (individual or family) resources, the severity of the limitation or disease, the time consumption and psychological burden of care, the infrastructural situation in the residential area, and the normative, cultural, and other individual beliefs and concerns of all involved persons. Up to now, there has been a lack of studies that investigate these factors on the county level; however, Kreft [13] concluded that the risk of long-term care is higher in counties where there are more deprived households.

#### Time trends in care need

We identified six studies published since 2001 which analyze trends in care need in Germany (Table 2). The review indicates an inconsistent picture of the trends in health in the last decades. Half (three) of the selected studies found a compression of long-term care [25–27]. The results of two studies can be interpreted as evidence for the dynamic equilibrium hypothesis [28, 29]. Only one study [30] found a relative expansion.

**Table 2:** Selection of studies investigating the health scenarios in Germany, publication year 2001 through 2015

Study	Type of health	Ages	Country/region	Time	Results	Regional comparison	Type of method
[25]	Long-term care (in general; incidence)	All ages	Germany	1998–2006	(Slight) **Compression**	no	Age standardized prevalence, incidence estimations
[26]	Long-term care (in general and severe)	All ages	North Rhine-Westfalia/Germany	1999–2005	Compression	no	Sullivan method
[27]	Long-term care (transition rates)	All ages	Berlin/Germany	2000–2009	**Compression** (but: policy influence assumed)	no	Transition models
[28]	Long-term care (by severity)	All ages	Germany	1999–2007	Dynamic equilibrium (Expansion for all types and stability for severe types of care)	Federal states and 3 regions	Sullivan method, standardized morbidity ratios
[29]	Long-term care (by severity of disability)	60+	Germany	1999–2008	Dynamic Equilibrium (Expansion for all types and stability for severe types)	no	Sullivan method
[30]	Long-term care (in general)	60+	Germany	1999–2005	**Relative expansion**	no	Sullivan method

*Note*: Words in **bold** letters indicate that the results are interpreted with a direct link to the health scenarios

These findings for Germany match the findings for other European countries and the United States (see [31–33] for reviews). The three reviews give evidence for different trends by severity of a health problem and indicate a dynamic equilibrium with expansion in mild health problems and stability or compression in severe disability. However, the choice of the health indicator (e.g., incidence, prevalence, or composed measures), the characteristics of the population under study (e.g., age groups, inclusion of institutionalized persons), the choice of the time perspective (due to societal and medical changes), and the design of the data (survey or administrative) affect the comparability of the findings of the studies.

The studies of Hackmann and Moog [25] and Häcker and Hackmann [27] used different methods and data than the other studies that applied the Sullivan method. While Hackmann and Moog [25] estimated the age standardized prevalence of care need by using 2004/06 data from the German Ministry of Health and explored incidence rates based on the (arbitrary) assumption of a stable internal age structure, Häcker and Hackmann [27] computed a transition model that used individual level data for SLTC-recipients in Berlin, a highly urbanized German region. Thus, the findings are not comparable to our study. In contrast, Pinheiro and Krämer [26], Pattloch [28], Unger and colleagues [29], and Hoffmann and Nachtmann [30] used the Sullivan method and an administrative data source (the SLTC census, except Unger and colleagues [29], who used health claims data from the Gmündener Ersatzkasse/GEK). Results of these studies indicate an expansion/relative expansion of long-term care in general and stability in severe types of care (dynamic equilibrium). Only Pinheiro and Krämer [26] found a compression in general and severe care need, which may be explained by the above-average decrease in prevalence of care between 1999 and 2007 (see Pattloch [28]:153).

In sum, this study has two objectives. First, we investigate the trends in LE, CFLY, CLY, and HR on the level of counties and classify them according to the theoretical health scenarios: expansion, compression, and stability. Second, we explore whether the changes in mortality or in morbidity are the driving factors behind experiencing a specific health scenario. We examine this by decomposing the county-specific CFLY and CLY trends into the effects of morbidity and mortality.

***Hypothesis 1:*** Based on the findings of previous studies [12, 13, 15, 20, 34–48] we hypothesize that there are county-specific differences in the trends of the health indicators which may lead to a heterogeneous pattern of the health scenarios. Given the remarkable increase in life expectancy of East Germany since reunification, it is not obvious whether the distribution of county-specific health scenarios is similar to West Germany. In addition, there are large subnational differences in the patterns of selected chronic diseases, their direct (e.g., smoking and obesity) and indirect (e.g., socioeconomic deprivation) risk factors, the medical infrastructure, and the major causes of death [13, 15, 18–23] These health(−relevant) regional characteristics combined with the multiple factors that affect the decision to apply for SLTC may result in different health scenarios.

***Hypothesis 2:*** However, based on the previous hypothesis and on earlier research that points towards a compression or equilibrium scenario [30, 49], we expect that this is also true in most – but not all – counties.

***Hypothesis 3:*** Turning to the contributions of the mortality and morbidity effects to the health scenarios, we do not have a specific hypothesis. A priori it is not obvious whether the same factor drives both the absolute changes in years of life with and without care need, and the resulting health scenarios. The reason for this is that the health scenarios are the result of interfering developments in the three distinct indicators CFLY, CLY, and LE. However, a decomposition analysis of trends in ADL among the French population aged 65 and above concluded that the compression found from 2004 to 2008 was predominantly caused by the change in the disability component rather than in the mortality component [50]. Whether this is also true for Germany is not clear.

## Data and methods

### Data

This study is based on the German Statutory Long-Term Care (SLTC) Censuses for the years 2001, 2003, 2005, 2007, and 2009. The SLTC Census is an official mandatory register of all long-term informal and formal care and care allowance receivers living in private households and institutions in Germany. The register is updated every two years and covers more than 2 million recipients of long-term care benefits as defined by the German Social Code Book XI. The register includes individual level information about sex, age, year of observation, care level (level 1 to 3/case of hardship), and the official ID of the residential county (NUTS 3 level) on December 31st of each year; no additional socioeconomic or demographic information is available. We aggregated the individual micro data by 5-year age groups (65–69, 70–74, 75–79,80–84, 85+), by sex, by year, by county, and by care level (level 1+ versus level 2+).

As participation is mandatory, the SLTC Census is not biased by non-response. Another advantage is the adequately high number of persons in need of care at the county level (Additional file 1: Table S1). To ensure data privacy, we use the total sample via remote access by the Research Data Centres of the Statistical Offices of the Federation and the Länder.

We combine the aggregated SLTC Census data with the vital data (population and death counts) of the official regional database of the National Statistical Office. Two problems with the data occur in the data management process.

First, the highest age group in the county-specific population statistics in 2001 is 75+, while in the other years there is a disaggregation in 5-year age groups until age 85 + .^1^ Thus, we estimated the population for the 5-year age groups by using available data for 2003–2009 and by assuming a constant change of the population shares within persons at age 75+ by sexes and counties from 2001 to 2009. We use an extrapolation method to estimate the population at the age groups 75–79, 80–84, and 85 and older in 2001.^2^

Second, in the observation period, two large – Saxony-Anhalt in 2007 and Saxony in 2008 – and two small reforms – Hanover in 2001 and Aachen in 2009 – of the counties were carried out. Most of these reforms were fusions of counties, which are unproblematic in terms of data management. For these counties, the data of the affiliated counties are pooled. For six counties in Saxony-Anhalt^3^ the reform of the counties fundamentally changed the geographical entities, which requires a more complex data management strategy. We choose an allocation of death counts and of the number of care receivers by using overall population based weights.^4^ The underlying assumption of this strategy is that the deaths and the persons in need of care are equally distributed in area of the counties and are not clustered in specific parts within a county.

### Care need

The care levels represent the intensity of restrictions in basic and instrumental activities of daily living (ADL and IADL) over a longer period. They are separated by the frequency and the time consumption of care assistance by non-professionals: persons with care level 1 need assistance at least once a day that takes more than 45 min for essential personal care and at least 90 min in total for general help; persons with care level 2 and higher need assistance for at least three times a day that takes 120 min or longer for essential personal care and at least 180 min in total for general help. The intensity of care is specified during a substantial home examination by members of the German medical service of health insurance [51].

As only official registered care need is used as the health outcome, there may be undercoverage of care need in general due to a lack of knowledge or high barriers of entry – for example, for persons with a migration background. However, there could also be differences (illegal, therefore hidden) in the evaluation process of the care level, as lobbyism towards the medical services and the financial resources of the insurance agencies may vary within Germany. In addition, it can be assumed that there is also a continuing (perhaps policy driven) change of assessment of the potential beneficiaries by the medical services in the observation period [27]. Further limitations are potential county-specific differences between East and West German counties in terms of individual acceptance of social benefits, as well as socioeconomic differences in terms of private financial resources to compensate public benefits.

### Methods

#### Sullivan method

We calculated care need-free life years (CFLY) and defined care need in terms of receiving financial and/or personnel support from the German SLTC insurance. Hereafter, the words long-term care, disability, and care need are used synonymously. The CFLY estimation is based on the Sullivan method [52] and on the Chiang method [53] for life expectancy (LE). We computed prevalence rates of care need separated by sex, age group (‘under 60’, ‘60–69’, ‘70–74’, ‘75–79’, ‘80–84’, ‘85+’), year of observation (2001, 2003, 2005, 2007, 2009), county, and care level.

We use two definitions of care need: all types of care (levels 1–3) versus severe type of care (level 2 and 3/case of hardship). The life years with care need (CLY) are calculated as the remaining total LE minus CFLY. The health ratio (HR) is the proportion of CFLY in total remaining LE. We estimate yearly LE, CFLY, CLY, and HR for both sexes and care levels, and for all 412 German counties within the borders of 2009. To reduce random fluctuations in the county’s death rates, we use pooled 3-year death counts for the estimation of the abridged life tables.

#### Trend analysis

In the first stage, we separately examine the temporal changes in the general level of the seven indicators (LE, CFLY_any_, CFLY_severe_, CLY_any_, CLY_severe_, HR_any_, HR_severe_). We combine the information of the indicators to classify the counties into the five established health scenarios plus regions with decreasing life expectancy (Table 1). To minimize random fluctuations in the indicators, we used pooled data for the two starting years (2001/2003) and the two final years (2007/2009). We define the trends as the estimated value in the last two years subtracting the estimated value in the first two years. An increase (a decrease) in an indicator is defined as a positive (negative) change, while, since continuous variables are used, stability is defined as an indicator change between −0.1 and +0.1.

#### Decomposition

In the second stage, we decompose county-specific CFLY_any_, CFLY_severe_, CLY_any_, and CLY_severe_ into the effects of morbidity and mortality, which measures the life years lost or gained due to changes in mortality or morbidity rates. We use the decomposition method by Nusselder and Looman [14], which is an extension of the Arriaga method [54]. We compare sex-specific CFLY and CLY in 2001/03 (t_1_) versus 2007/09 (t_2_). The change in the number of person-years with care need (CLY) for a particular county, sex, and care level is measured by

1 $$ {}_iCL{Y}_x{=}_{\mathrm{i}}{\mathrm{Mort}}_{\Delta \mathrm{CLY},\ \mathrm{x}}{+}_{\mathrm{i}}{\mathrm{Morb}}_{\Delta \mathrm{CLY},\ \mathrm{x}}=\left(\frac{{}_ipre{v}_{x_{t1}}{+}_ipre{v}_{x_{t2}}}{2}\right)\times {\varDelta}_i{L}_x+\left(\frac{{}_i{L}_{x_{t1}}{+}_i{L}_{x_{t2}}}{2}\right)\times {\varDelta}_ipre{v}_x, $$

where x depicts age, i the length of the age interval, _i_L_x_ the product of person-years lived, and _i_prev_x_ the prevalence of care need. The number of person-years without care need (CFLY) is decomposed in the same manner.

### Multinomial logistic regression

In the third stage, we estimated multinomial logistic regression models to analyze the association of the morbidity and mortality effects with the health scenarios. We used the three theoretical health scenarios (expansion, stability, compression) rather than the five categories presented in Table 1 due to the low number of counties in some of the categories. The explanatory variables are the mean centred morbidity (Morb) and mortality effects in CFLY (Mort_ΔCFLY_) and in CLY (Mort_ΔCLY_), which are measured in life days. To account for county-specific uncertainty of CFLY_any_ and CFLY_severe_ estimation, we use weighted regression models.^5^

The regression model for persons aged 65+ and of a particular sex and care level is defined by

2 $$ Logi{t}_{i,j}= log\frac{Pr\left({Y}_i=j\right)}{Pr\left({Y}_i=j\hbox{'}\right)}={\alpha}_j+{\beta}_{1,j}{\mathrm{Morb}}_i+{\beta}_{2,j}{\mathrm{Mort}}_{\Delta \mathrm{CFLY},i}+{\beta}_{3,j}{\mathrm{Mort}}_{\Delta \mathrm{CLY},i}, $$

where i depicts the county, j is the particular health scenario (stability or compression), j’ is the reference health scenario (expansion), α is the intercept, and the βs are the estimated coefficients.

All calculations are performed using Stata 12.1 and a decomposition tool programmed in R by WJ Nusselder and CWN Looman.^6^ The results are given as relative risk ratios (RRR) on the chance of being a “stability” or a “compression” county versus being an “expansion” county (reference) for both sexes aged 65+, and for any/severe care level.

## Results

In the period from 2001 to 2009, the number of persons in care need has increased from 2.04 to 2.34 million. Thus, the raw care need prevalence is about 2.5 % in 2001 and 2.9 % in 2009. Of these, nearly 50 % have care level 1 (2001: 0.89 million; 2009: 1.25 million persons). The majority are female (2001: 1.40 million; 2009: 1.57 million); however, the increase between 2001 and 2009 is higher for males (+20 %) than for females (+12 %). About 81 % (2001) respectively 83 % (2009) are 65 years and older and the total increase is solely due to these ages (+18 %). On the contrary, the absolute number of persons younger than 65 is nearly stable (+0.09 %) over time.

### Trends according to the five health scenarios

Taking the unweighted mean over all counties, remaining LE, CFLY_any_, and CFLY_severe_ have been continuously increasing for both sexes (Table 3). CLY_any_ also increased, while there was no significant time trend for CLY_severe_. An analysis of the time trends in HR – separated by men and women and by severity of care need – confirms the findings. The proportion of life years free from any care level (HR_any_) decreased, while the proportion of life years free from severe care level (HR_severe_) remained stable or even increased slightly.

**Table 3:** Level (measured by the county-level mean) and spatial dispersion (measured by interquartile range; IQR) of life expectancy total, with and without any care level and with and without severe care level and the health ratios, men and women at age 65+, 2001–2009

		Men					Women				
		2001	2003	2005	2007	2009	2001	2003	2005	2007	2009
LE	Mean	15.97	16.47	16.75	17.21	17.43	19.26	19.60	19.98	20.40	20.55
		[15.90–16.03]	[16.40–16.54]	[16.68–16.82]	[17.14–17.28]	[17.36–17.51]	[19.20–19.32]	[19.54–19.65]	[19.92–20.03]	[20.35–20.45]	[20.50–20.61]
	IQR	0.898	0.906	0.960	0.993	1.014	0.900	0.858	0.815	0.796	0.808
CFLE_any_	Mean	14.39	14.85	15.03	15.39	15.60	16.22	16.53	16.76	17.03	17.17
		[14.32–14.46]	[14.78–14.93]	[14.95–15.10]	[15.31–15.47]	[15.51–15.69]	[16.14–16.29]	[16.45–16.60]	[16.68–16.84]	[16.95–17.11]	[17.09–17.26]
	IQR	1.053	1.144	1.068	1.149	1.157	1.049	1.046	1.107	1.193	1.231
CLY_any_	Mean	1.58	1.62	1.72	1.82	1.83	3.05	3.07	3.22	3.37	3.38
		[1.56–1.60]	[1.59–1.64]	[1.70–1.74]	[1.79–1.85]	[1.81–1.86]	[3.01–3.08]	[3.03–3.11]	[3.18–3.26]	[3.32–3.42]	[3.33–3.44]
	IQR	0.293	0.312	0.355	0.375	0.373	0.520	0.552	0.569	0.657	0.750
HR_any_	Mean	90.08	90.16	89.70	89.40	89.43	84.17	84.32	83.86	83.45	83.51
		[89.93–90.22]	[90.01–90.31]	[89.54–89.86]	[89.22–89.58]	[89.25–89.62]	[83.95–84.38]	[84.10–84.54]	[83.63–84.09]	[83.19–83.71]	[83.24–83.79]
	IQR	1.880	1.940	2.327	2.529	2.462	3.076	2.959	3.133	3.466	3.849
CFLE_severe_	Mean	15.14	15.65	15.89	16.32	16.58	17.67	18.03	18.35	18.75	18.97
		[15.08–15.21]	[15.59–15.72]	[15.82–15.96]	[16.25–16.40]	[16.50–16.65]	[17.60–17.73]	[17.97–18.09]	[18.28–18.41]	[18.69–18.81]	[18.90–19.03]
	IQR	0.956	1.055	1.043	1.081	1.082	0.968	0.931	0.909	0.914	0.907
CLY_severe_	Mean	0.82	0.82	0.86	0.88	0.86	1.59	1.57	1.63	1.65	1.59
		[0.81–0.84]	[0.80–0.83]	[0.87–0.87]	[0.87–0.90]	[0.87–0.87]	[1.57–1.62]	[1.55–1.59]	[1.61–1.66]	[1.62–1.68]	[1.56–1.62]
	IQR	0.200	0.196	0.210	0.220	0.193	0.279	0.311	0.344	0.345	0.336
HR_severe_	Mean	94.84	95.04	94.86	94.84	95.06	91.72	91.98	91.81	91.89	92.26
		[94.75–94.92]	[94.95–95.13]	[94.76–94.96]	[94.74–94.95]	[94.97–95.16]	[91.60–91.85]	[91.86–92.11]	[91.68–91.94]	[91.75–92.03]	[92.13–92.39]
	IQR	1.210	1.247	1.394	1.453	1.208	1.606	1.668	1.747	1.850	1.789

*Source*: Statistical Offices of the Federation and the Länder, Statutory Long-Term Care Censuses 2001–2009 & Regional database (2013); author’s calculation

In detail, mean male LE increased from 15.97 to 17.43 years and mean female LE rose from 19.26 to 20.55 years. Thus, the gain was higher for men (0.18 life years per annum) than for women (0.16 life years per annum). While the spatial variation in LE increased for men (from interquartile range IQR = 0.898 to 1.014), that of women decreased (from 0.900 to 0.808) in this period. CFLY shows an increase in both, CFLY_any_ and CFLY_severe_. Mean CFLY_any_ rose from 14.39 years (IQR = 1.053) to 15.60 (IQR = 1.157) in men and from 16.22 (IQR = 1.049) to 17.17 years (IQR = 1.231) in women. CFLY_severe_ has increased from 15.14 (IQR = 0.956) to 16.58 years (IQR = 1.082) in men and from 17.67 (IQR = 0.968) to 18.97 (IQR = 0.907) in women. Thus, the increase in CFLE_severe_ is higher than in CFLE_any_. Mean CLY_any_ of males increased from 1.58 to 1.83 years and those of females from 3.05 to 3.38 years. In contrast, male CLY_severe_ stagnated at around 0.85 and female CLY_severe_ at around 1.61 years.

The trends are weakly correlated with the starting level in 2001/2003. While in the case of male LE, there is no association of the level with the trend component (Pearson correlation = -0.07, *p* > 0.1), the increase in female LE is lower in counties with a high LE starting level (-0.33, *p* < 0.001). For CFLY, there are inconsistent associations. There is a weak positive correlation in case of CFLY_any_ in men (0.17, *p* < 0.001), but no correlations in male CFLY_severe_ and in female CFLY_any_ (both 0.06, *p* > 0.1). However, we did find a weak negative correlation in female CFLY_severe_ (-0.18, *p* < 0.001). In CLY, there are no correlations in CLY_any_ (men: -0.07; women: 0.03, both *p* > 0.1) and weak negative correlations in CLY_severe_ (men: -0.20, *p* < 0.001; women: -0.11, *p* = 0.03).

We spatially plot selected variants by the starting level in 2001/2003 and by the trends up to 2007/2009, and detect notable clusters of counties with very favorable and very unfavorable combinations. In case of LE, CFLY, and HR, unfavorable combinations are defined as a low starting level and the lowest (more than one standard deviation below the county-level mean) change over the period. In the case of CLY, in contrast, unfavorable combinations are defined as a high starting level and the highest (more than one standard deviation above the county-level mean) change over the period. For the trends in LE, CFLY, CLY, and HR, there is a slight but consistent gradient between the most disadvantaged counties in the North, Middle and East of Germany – including eastern Bavaria – and the most advantaged counties in the South and West (Additional file 1: Table S2–S5).

By combining the trends in the various indictors into the health scenarios for all of Germany, we find a relative expansion for any care level for both sexes, but a stable trend in severe care level of males and a relative compression in severe care level of females.

**Fig. 1:**
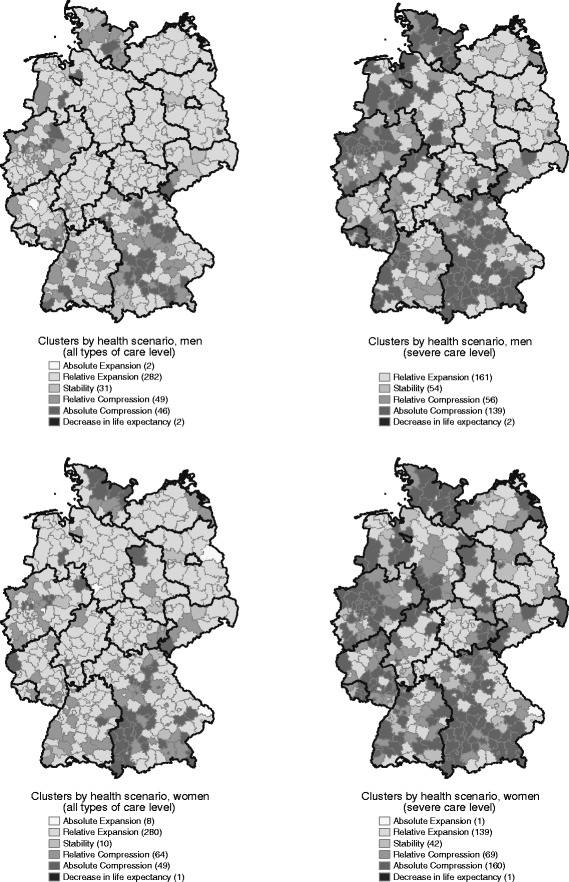
Clusters by health scenarios for any care level and for severe care level, men and women, age 65+, 2001/03–2007/09. Source: Statistical Offices of the Federation and the Länder, Statutory Long-Term Care Censuses 2001-2009 & Regional database (2013); author’s calculations and mapping

In contrast to the picture of a nationwide consistent trend, the health scenario classification on level of counties reveals a high subnational heterogeneity (Fig. 1). Obviously, there is no clear east–west or north–south gap, but a high divergence within the particular federal states. Nevertheless, in case of any care level, the majority of the counties have experienced a relative expansion. Almost every county in the federal states Lower Saxony, Hesse, northern Rhineland-Palatinate, northern and eastern Bavaria, and the majority of the East German counties are in the relative expansion cluster. The highest spatial heterogeneity can be stated for Schleswig-Holstein, North Rhine-Westphalia, Baden-Württemberg, and Saxony. The general spatial pattern of the health scenarios is consistent for men and women (Spearmans rho = 0.60); however there are some exceptions (some counties in Schleswig-Holstein, Saxony-Anhalt, North Rhine-Westphalia, and Bavaria).

In terms of trends in severe care levels, the number of counties experiencing an expansion is lower than in case of any care level. As a consequence, there are comparatively more counties classified as counties with relative and absolute compression. However, there is a higher level of bipolarization with counties experiencing a relative expansion and counties experiencing a compression for males than for females. This is the explanation for the stable trend for males on the national level.

Looking at any and severe care level simultaneously, the majority of counties show either an expansion in both care levels or a dynamic equilibrium, including the shift from more to less severe levels as defined by Manton [7]. In case of men, we classify 161 out of 412 counties into these two groups and, in case of women, 137 out of 412 counties. In contrast, 93 counties (men) and 108 counties (women), respectively, experienced a compression in both levels. An expansion/equilibrium in any care level combined with a compression/equilibrium in severe care level is detected in 154 counties (men) respectively 161 counties (women).^7^

### Decomposition of the trends - the role of morbidity and mortality effects

Over all counties and for both sexes, the mortality trends have the highest effect on CFLY and CLY in absolute values. On average, from 81 up to 92 % of the increases in CFLY are caused by mortality reductions in CFLY and only 8 to 19 % by morbidity changes (Table 4). Mean Mort_ΔCLY_ is low, but the overall mean morbidity effect is even lower. The proportion of Mort_ΔCLY_ ranges between 135 and 656 %. Thus, survival improvements are of higher impact on CLY trend than on the trends in CFLY, especially in case of trends in CLY_severe_. The spatial mapping of the trends of the mortality and morbidity effects shows high heterogeneity and no clear clusters (Additional file 1: Table S6–S8).

**Table 4:** Mean absolute and relative change in life expectancy, care need-free life years and life years with care need at age 65+ by sex and care level, 2001/03–2007/09

		Mean change in			CFLY change due to		CLY change due to	
		LE	CFLY	CLY	Mortality	Morbidity	Mortality	Morbidity
Any Care Level	Men	1.179	0.965	0.214	0.890	0.075	0.289	−0.075
					92 %	8 %	135 %	−35 %
	Women	1.137	0.811	0.326	0.668	0.142	0.468	−0.142
					82 %	18 %	144 %	−44 %
Severe Care Level	Men	1.179	1.139	0.040	1.038	0.101	0.142	−0.101
					91 %	9 %	356 %	−253 %
	Women	1.137	1.100	0.037	0.893	0.207	0.244	−0.207
					81 %	19 %	667 %	−565 %

*Note*: All means are weighted by 1/∑_i = 2001/03_^2007/09^(σ^2^(CFLY_i_))

*Source*: Statistical Offices of the Federation and the Länder, Statutory Long-Term Care Censuses 2001–2009 & Regional database (2013); author’s calculations

The results of the decomposition reveal a high variability in terms of combinations of the morbidity and the two mortality effects. We define the categories “low” (“high”) as values less (more) than one standard deviation below (above) the mean, and “medium” as values close to the mean. By definition, most counties have medium morbidity and mortality effects. These counties are mostly expansion counties in case of any care level and mostly compression counties in case of severe care level.

Some combinations do not exist. These are the combinations of a low mortality effect in CLY trend (Mort_ΔCLY_) and a high mortality effect in CFLY trend (Mort_ΔCFLY_) – the most favorable trend – and vice versa – the most unfavorable trend.

**Fig. 2:**
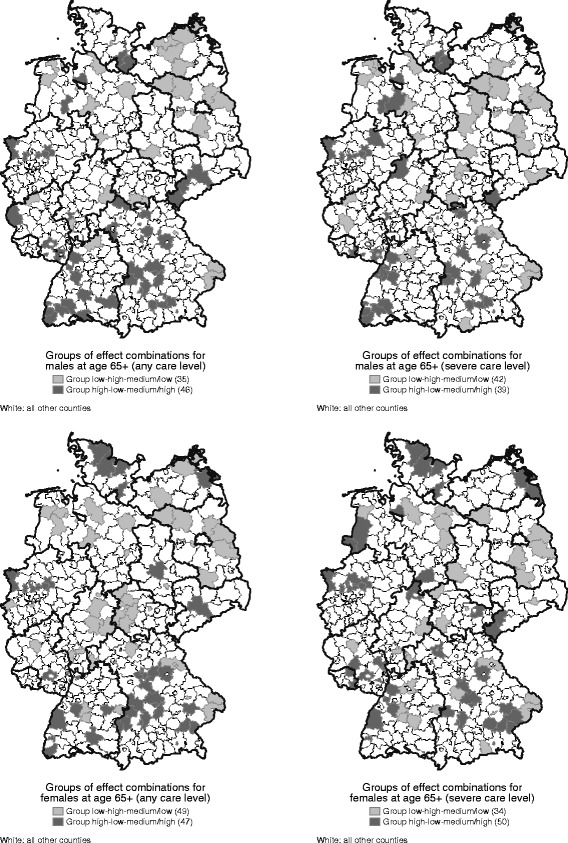
Groups of effect combinations by sex and severity of care level. Source: Statistical Offices of the Federation and the Länder, Statutory Long-Term Care Censuses 2001-2009 & Regional database (2013); author's calculations and mapping

The two counties Greifswald and Barnim in northeast Germany show the most unfavorable trends and are both experiencing an expansion in any and severe care need. Almost every county with a high morbidity effect is a compression county, while nearly all counties with low morbidity effects are expansion counties. The counties with the second most unfavorable trend (“low morbidity – high Mort_ΔCLY_ – medium Mort_ΔCFLY_”) are counties in East Germany, in Lower Saxony, and Eastern Bavaria and for females (any care level), these are central Germany (Fig. 2). The counties with the most favorable trends are located in the South German regions and, for females, in the very north of Schleswig-Holstein. These counties are merely compression counties.

More insight can be gained from the association of the morbidity effects with each of the two mortality effects. We estimated bivariate linear regressions for each combination of the three effects differentiated by compression and expansion counties (Additional file 1: Table S9–S11). In terms of CFLY, both the morbidity and the mortality effect add up to additional healthy life years. Counties where morbidity improvements lead to large gains in CFLY are merely compression counties. This association is weakly dependent on Mort_ΔCFLY_, as indicated by the weak positive slope of the regression line (slopes = [0.040; 0.284]). The slope is similar in compression and expansion counties. The weak positive association is true for both sexes as well as for any and severe care level. In terms of CLY, the morbidity effect must be larger than the mortality effect in terms of compression countries. Thus, the correlation of Mort_ΔCLY_ and morbidity effects is higher in the compression counties (slopes = [0.537; 0.632]) than in the expansion counties (slopes = [0.176; 0.391]).

Turning to the multinomial regression, we find that the morbidity effect has the highest impact on the health scenarios (Table 5). An increase of CFLY due to reductions in prevalence leads to a massively higher chance of being a dynamic equilibrium county (RRR = [1.271;2.679]) and a compression county (RRR = [1.640;9.893]). Additionally, a gain in Mort_ΔCFLY_ also results in a negligibly higher chance of experiencing stability (RRR = [1.012;1.052]) or a compression (RRR = [1.011;1.098]). The influence of Mort_ΔCFLY_ is statistically significant for males only. On the contrary, an increase in Mort_ΔCLY_ leads to a significant decrease in the chance of a county to experience stability (RRR = [0.494;0.851]) or a compression (RRR = [0.205;0.722]).

**Table 5:** Results of the four multinomial regression models for males and females at age 65+ by care level, mean centred morbidity and mortality effects are measured in change in life days

	Sex	Covariates (Health scenarios)	Cases (Counties)	Mortality		Mortality		Morbidity			
				effect in CLY		effect in CFLE		effect in CFLE		Pseudo	Miss-
				RRR	*p*-value	RRR	*p*-value	RRR	*p*-value	R^2^	sings
Any Care Level	Males	Ref: Expansion	284	1		1		1			
		Stability	31	0.531	<0.001	1.052	0.001	2.341	<0.001	0.95	2
		Compression	95	0.251	<0.001	1.098	<0.001	5.980	<0.001		
	Females	Ref: Expansion	288	1		1		1			
		Stability	10	0.851	<0.001	1.021	0.064	1.271	<0.001	0.91	1
		Compression	113	0.722	<0.001	1.011	0.442	1.640	<0.001		
Severe Care Level	Males	Ref: Expansion	161	1		1		1			
		Stability	54	0.494	0.001	1.015	0.027	2.679	0.004	0.95	2
		Compression	195	0.230	<0.001	1.035	<0.001	7.464	<0.001		
	Females	Ref: Expansion	140	1		1		1			
		Stability	42	0.613	<0.001	1.012	0.130	1.964	<0.001	0.95	1
		Compression	229	0.205	<0.001	1.017	0.115	9.893	<0.001		

*Note*: counties are weighted by 1/∑_i = 2001/03_^2007/09^(σ^2^(CFLY_i_))

*Source*: Statistical Offices of the Federation and the Länder, Statutory Long-Term Care Censuses 2001–2009 & Regional database (2013); author’s calculations

## Discussion

To our knowledge, this is one of the first studies that explores trends in life years with and without care need and in the resulting health scenarios on a small-area level. Our study confirms that there is high county-level heterogeneity in the trends of the health indicators and in the health scenarios.

Turning to our first research question, the stratified investigation of the trends by care level shows that there are different care need trends in any and in severe care level. While the majority of counties experience a relative expansion of any care level, the mean remaining life span with a severe care level shows stability or compression. For both sexes, the majority of the counties experience a similar health scenario as the whole country. One exception is males with severe care level. For those, the aggregation of the expansion and compression counties to the total country level leads to the wrong conclusion of a stable trend. By combining these trends, our findings confirm the extended theory of dynamic equilibrium that assumes an expansion of morbidity with a shift from severe to moderate types of morbidity [7]. Thus, our conclusions are consistent with previous findings [28, 29, 56, 57].

The diversity in the trends in the health indicators and the notable subnational heterogeneity in terms of the health scenarios cause a disparity in the level of current and future challenges in public health and in social policy according to financial, infrastructural, sociohumanitarian, and welfare state aspects. In the most disadvantaged situation are those counties where the population shows an absolute expansion of care need. The most favorable position is found in counties experiencing an absolute compression. In contrast to the spatial pattern of LE [15], there was no indication for a clear northeast versus southwest gap in both, the health scenarios and the sole trends in the particular indicators.

These findings are strong evidence that there are profound differences between quantity (life expectancy) and quality (care-free life years, health ratio) of life time in the longitudinal trend of the indicators. The classification of the counties by starting level and by trend of the health indicators observed over time unfolds the expected spatial pattern showing counties with unfavorable levels and trends in the North, East and Middle of Germany versus counties with favorable levels and trends in the South and West. Hence, the vanguard counties increased their lead over the rearguard counties in 2001–2009. Furthermore, our study shows that through all counties the higher the level of female LE, of female CFLY_severe,_ and of CLY_severe_ for both sexes in 2001/2003, the lower the changes until 2007/2009. This is an indication for an upper level of these indicators. Only for male CFLY does there seem to be an accelerating process of increase which indicates a much higher potential of gains in life years without care need in future.

We explain these findings by a complex interference of different epidemiological processes. On the one hand, regional disparities are expected to be the result of divergent historical regional developments and current regional conditions that have joint interfering, mediating, and suppressing regional specific effects. Those can be period and/or cohort effects on the behavior, the psychosocial capacity, and the material situation over the life course (timing and duration) of the individuals [58] that in turn have an indirect effect on the total population’s composition. On the other hand, the disparities are the direct result of different compositions of the county’s population due to the continuous processes of selectivity because of regional specific trends in mortality and migration [59–61].

Turning to our second research question, where we did not have a specific a priori hypothesis, we find that in absolute terms, by far the majority of the absolute increase in disability-free life years and disabled life years is caused by the increase in the survival of the non-disabled and disabled. In other words, the decrease of mortality rates is decisive for the number of additional years with and without care need. In terms of the health scenarios, however, the morbidity effects, respectively the trends in the prevalence of care need, are the decisive drivers of the chance to experience a compression or an expansion. The mortality effects on the change in disabled life years and on the change in disability-free life years are of much lower importance. This can be stated for both sexes and for any and severe care levels. Thus, slight absolute changes in the prevalence rates of care need have a very high impact on a county’s health scenario. These findings confirm the results of Cambois and colleagues [50].

One explanation for the differences between any and severe care level is that the findings are evidence for the dynamic equilibrium theory assuming a shift from severe to moderate care need. Improvements in health services, a higher awareness of health problems, increased medical knowledge, earlier diagnostics, and better and less risky surgical and medical interventions lead to an enlargement of life time with (severe) physical and mental limitations [33, 62]. Another explanation for the expansion is that the increase is a result of a changed behavior of the elderly in terms of acceptance of social benefits, which can be described as a shift from a “gratitude” generation to a “demand” generation. One indication for this argument is the disproportional increase in the initial health evaluations by the medical services of the STLC insurance. Between 2001 and 2009, there was a gain of 23 % [63], while the population at age 65+ increased only by 9 % [64]. The different trends of the two care level groups may be only the result of a higher restriction in legal acceptance assuming that the higher the care level, the more intensive the medical evaluations and the higher the legal and individual barriers. Indirect evidence for the higher restrictions are the decisions of the re-evaluations of more than 40 % of the care receivers conducted annually by the medical services. For example, in 2006, 45.8 % (outpatient) and 69.7 % (inpatient) of the re-evaluated persons in care level 1 were upgraded to a higher care level, while it was only 36.6 and 56.3 % respectively of the persons in care level 2 [65].

Our study has profound strengths. One advantage is the large number of persons included in the STLC censuses, allowing us to investigate trends on subnational level. Because the census is mandatory for all private and public STLC beneficiaries, from an administrative and health care planning point of view, the data are not biased by missing records or problems of loss due to follow-up. The health outcome itself is another advantage, because it is an objective, nationally standardized evaluation by medical experts of the health insurance companies. A third strength is that we assume only a marginal bias due to cultural differences in the definition of care need, as all SLTC regulations are harmonized and binding for all counties. We used the established healthy life years measure that allows comparisons of the health situation even for small populations and only if cross-sectional data for the individuals is available. The use of the advanced method of decomposition by Nusselder and Looman [14] provides deeper insights in the complex interactions of changes in the subnational mortality and morbidity patterns and how these affect health scenarios in Germany. The longitudinal design of the data of the counties is an advantage in many ways; e.g., to investigate the stepwise changes and to compare baseline level with time trends.

However, there are also limitations. First, because only aggregated data was accessible, we are not able to identify whether the disparities are the result of changes in the population’s composition due to 1) (health-related) selective migration and selective mortality or are 2) causally related to the life time accumulation or coping mechanisms on the residential hazardous conditions of the individuals. Second, there is also the restriction that the design of the study did not allow us to reveal whether specific cohort or period effects in care need cause a higher magnitude and a higher pace of the county-specific changes. Third, a limitation caused by the design of the study may be the definition and the restrictions in the temporal and cross-county comparability of the health indicator. Fourth, another potential bias may be the quality of the data for the sex- and age-specific population in the counties. Because the population information (unlike birth and death statistics) is not based on registers or a census, but rather on extrapolation estimations, unregistered in- and out-migration may lead to a bias that is expected to be higher at the oldest age groups [66].^8^ Post-analyses, however, show that the bias is marginal. Fifth, registration problems of the SLTC census for the years prior to 2009 may affect the results. Until 2008, an unknown number of persons with semi-inpatient care were double-counted, leading to an overcoverage of persons with care need [67]. Because the share of persons in semi-inpatient care to all persons with care need is very low – in Germany in total about 2 % [67] – this bias is also expected to be marginal. Sixth, yet another limitation is that we do not have county-specific population data by age groups beyond age 85; thus, we are not able to analyze the trends in the internal composition and in the regional disparities at the highest age groups. The prevalence of care need at these ages, however, is very high and therefore regional disparities in CFLY and CLY may be underestimated. Seventh, methodological problems may be caused by using prevalence data with the Sullivan’s method instead of individual-level panel data about specific transitions in a multi-state model. Prevalence data overlook the duration of care need or the complexity of possible transitions, which leads to a large bias when the transition rates are highly fluctuable ([68]:86). One study ([28]:101), however, concluded that the limitations of the Sullivan method are acceptable for SLTC data, as the transition rates are assumed to be very stable (incidence/mortality) or very low (rehabilitation). Another problem with the Sullivan method is the dichotomization of the health outcome (with/without care need) which is a simplification of a complex morbidity continuum. We use the strategy of Pattloch [28] to face this problem by analyzing the trends in care need by different levels of severity.

## Conclusion

Our study shows a high diversity in care need challenges on level of counties in Germany. While some counties show very positive trends in terms of a compression of care need, others are confronted with a growing proportion of persons in care need living longer with disability. Overall, the shift from severe to moderate care need is a favorable development considering the financial and emotional burden for individuals and society.

Furthermore, our study detects that the place of residence is another important influence factor of the trends in care need. The study demonstrates that there is a complex interaction between trends in care need prevalence and mortality rates. Since we found that the prevalence is the main driver of the health scenarios, higher efforts are required to reduce the prevalence rates. This is of particular importance in counties in the north and the east of Germany that already have the highest share of persons in care need. In future research it will be important to investigate the trends in the new^9^ care level 0 and the causes of the diversity in the mortality and morbidity effects. Thus, one of the emerging questions is whether the specific living conditions in the counties and their changes over time are associated with the trends in care need and mortality. Previous studies [13, 44, 69–71] have found associations of regional characteristics with small-area health conditions, but studies about health trends are rare. Further investigations are needed to uncover the underlying mechanisms of health aging to understand and to deal with the challenges of an increasingly more heterogeneous aging society.

## Additional files

Additional file 1:
**Table S1.** Mid-year population, deaths, and number of persons with any and severe care level by sex at age 65+ in 2001, 2003, 2005, 2007, and 2009, Germany. **Table S2.** LE trend by county for men and women at age 65, 2001/03-2007/09. **Table S3.** CFLY trend by county and severity of care need for men and women at age 65, 2001/03-2007/09. **Table S4.** CLY trend by county and severity of care need for men and women at age 65, 2001/03-2007/09. **Table S5.** HR trend by county and severity of care need for men and women at age 65, 2001/03-2007/09 (PP=percentage points). **Table S6.** Decomposition results: Effects of mortality on the CFLY trend by county, sex and severity of disability for persons at age 65+, 2001/03-2007/09. **Table S7.** Decomposition results: Effects of mortality on the CLY trend by county, sex and severity of care need for persons at age 65+, 2001/03-2007/09. **Table S8.** Decomposition results: Effects of morbidity by county, sex and severity of care need for persons at age 65+, 2001/03-2007/09. **Table S9.** Scatterplots of morbidity effects and mortality effects on the CFLY trend at age 65+ by care level and sex (only counties with expansion or compression included), higher symbol size indicates a higher estimation precision/a lower uncertainty. **Table S10.** Scatterplots of morbidity effects and mortality effects on the CLY trend at age 65+ by care level and sex (only counties with expansion or compression included), higher symbol size indicates a higher estimation precision/a lower uncertainty. **Table S11.** Scatterplots of mortality effects on CFLY trend and mortality effects on CLY trend at age 65+ by care level and sex (only counties with expansion or compression included), higher symbol size indicates a higher estimation precision/a lower uncertainty. (DOCX 5.91 MB) Additional file 2: County-specific study results. (XLS 398 kb)

## Footnotes

1 An additional disaggregation of the population data at the ages 85–89, 90–95, and so on is not available on a county level due to data privacy laws.

2 For instance: If there is a %P_Men,85+,county 1,2003_(proportion of men at age 85+ to all men at age 75+ in county 1 in 2003) of 15 % and a %P_Men,85+,county 1,2005_ of 16 %, the estimation of %P_Men,85+,county 1,2001_ is 14 %.

3 The counties are Harz, Salzlandkreis, Jerichower Land, Anhalt-Bitterfeld, Wittenberg, and Dessau-Roßlau.

4 The overall population weights are based on the total population counts for those years 2001–2006 in which we have information for the old and the new regional entities. For each single year, the (positive or negative) difference between the population of the new and of the old (P_old_) entities equals the population that experienced an administrative change (P_change_). In the last step, the population weights are computed by P_change_ divided by P_old_.

5 The county- and sex-specific precision weights are computed by 1 dividing by the variance of CFLY_any_, respectively CFLY_severe_ at age 65 + . For further information for the calculation of the variance and the standard errors of CFLY, see [55].

6 The decomposition tool and the user guide are available on request (contact: w.nusselder@erasmusmc.nl)

7 The remaining 4 (men) or 6 (women) counties show inconsistent combinations for both levels or a LE decrease.

8 By comparing the extrapolation results and the results of the official population census in 2011, we show that the cumulated overestimation of the population 65+ in 2011 is only about 1.89 % in Germany as total and only 1.42 % in the unweighted median of all counties. Further, estimated partial CFLY, CLY, HR and LE for the ages 65–84 are highly correlated with those at age 65+ (correlations range from r(LE, females, 2007/09) = 0.86 to r(HR, any care, females, 2007/09) = 0.97)

9 Care level 0 was introduced in 2008 and covers persons with permanent significant limitations in everyday life competence, who have a need for assistance in the field of basic nursing and household assistance, but who do not yet fulfill the requirements for the qualification into the nursing care level 1.
