# Supplementary material for: Expansion or compression of long-term care in Germany between 2001 and 2009? A small-area decomposition study based on administrative health data
Source: Popul Health Metr. 2016 Jul 13;14:24. doi: 10.1186/s12963-016-0093-1 (PMC4944474; doi:10.1186/s12963-016-0093-1)
Supplement: Additional file 1: — Table S1. Mid-year population, deaths, and number of persons with any and severe care level by sex at age 65+ in 2001, 2003, 2005, 2007, and 2009, Germany. Table S2. LE trend by county for men and women at age 65, 2001/03-2007/09. Table S3. CFLY trend by county and severity of care need for men and women at age 65, 2001/03-2007/09. Table S4. CLY trend by county and severity of care need for men and women at age 65, 2001/03-2007/09. Table S5. HR trend by county and severity of care need for men and women at age 65, 2001/03-2007/09 (PP=percentage points). Table S6. Decomposition results: Effects of mortality on the CFLY trend by county, sex and severity of disability for persons at age 65+, 2001/03-2007/09. Table S7. Decomposition results: Effects of mortality on the CLY trend by county, sex and severity of care need for persons at age 65+, 2001/03-2007/09. Table S8. Decomposition results: Effects of morbidity by county, sex and severity of care need for persons at age 65+, 2001/03-2007/09. Table S9. Scatterplots of morbidity effects and mortality effects on the CFLY trend at age 65+ by care level and sex (only counties with expansion or compression included), higher symbol size indicates a higher estimation precision/a lower uncertainty. Table S10. Scatterplots of morbidity effects and mortality effects on the CLY trend at age 65+ by care level and sex (only counties with expansion or compression included), higher symbol size indicates a higher estimation precision/a lower uncertainty. Table S11. Scatterplots of mortality effects on CFLY trend and mortality effects on CLY trend at age 65+ by care level and sex (only counties with expansion or compression included), higher symbol size indicates a higher estimation precision/a lower uncertainty. (DOCX 5.91 MB) [file 12963_2016_93_MOESM1_ESM.docx]

S 1: Mid-year population, deaths, and number of persons with any and severe care level by sex at age 65+ in 2001, 2003, 2005, 2007, and 2009, Germany

Source: Statistical Offices of the Federation and the Länder, Statutory Long-Term Care Censuses 2001-2009 & Regional database (2014); author's calculations

S 2: LE trend by county for men and women at age 65, 2001/03-2007/09

|  |
| --- |

Source: Statistical Offices of the Federation and the Länder, Statutory Long-Term Care Censuses 2001-2009 & Regional database (2014); author's calculations

S 3: CFLY trend by county and severity of care need for men and women at age 65, 2001/03-2007/09

|    |
| --- |

Note: County-level mean is a mean weighted by $1/\sum_{i=2001/03}^{2007/09} \left( \text{σ² (CFLY}_{i}) \right)$

Source: Statistical Offices of the Federation and the Länder, Statutory Long-Term Care Censuses 2001-2009 & Regional database (2014); author's calculations and mapping

S 4: CLY trend by county and severity of care need for men and women at age 65, 2001/03-2007/09

|    |
| --- |

Note: County-level mean is a mean weighted by $1/\sum_{i=2001/03}^{2007/09} \left( \text{σ² (CFLY}_{i}) \right)$

Source: Statistical Offices of the Federation and the Länder, Statutory Long-Term Care Censuses 2001-2009 & Regional database (2014); author's calculations and mapping

S 5: HR trend by county and severity of care need for men and women at age 65, 2001/03-2007/09 (PP=percentage points)

|    |
| --- |

Note: County-level mean is a mean weighted by $1/\sum_{i=2001/03}^{2007/09} \left( \text{σ² (CFLY}_{i}) \right)$

Source: Statistical Offices of the Federation and the Länder, Statutory Long-Term Care Censuses 2001-2009 & Regional database (2014); author's calculations and mapping

S 6: Decomposition results: Effects of mortality on the CFLY trend by county, sex and severity of disability for persons at age 65+, 2001/03-2007/09

|    |
| --- |
| Note: County-level mean is a mean weighted by $1/\sum_{i=2001/03}^{2007/09} \left( \text{σ² (CFLY}_{i}) \right)$ |

Source: Statistical Offices of the Federation and the Länder, Statutory Long-Term Care Censuses 2001-2009 & Regional database (2014); author's calculations and mapping

S 7: Decomposition results: Effects of mortality on the CLY trend by county, sex and severity of care need for persons at age 65+, 2001/03-2007/09

|    |
| --- |
| Note: County-level mean is a mean weighted by $1/\sum_{i=2001/03}^{2007/09} \left( \text{σ² (CFLY}_{i}) \right)$ |

Source: Statistical Offices of the Federation and the Länder, Statutory Long-Term Care Censuses 2001-2009 & Regional database (2014); author's calculations and mapping

S 8: Decomposition results: Effects of morbidity by county, sex and severity of care need for persons at age 65+, 2001/03-2007/09

|    |
| --- |
| Note: county-level mean is a weighted mean by $1/\sum_{i=2001/03}^{2007/09} \left( \text{σ² (CFLY}_{i}) \right)$ |

Source: Statistical Offices of the Federation and the Länder, Statutory Long-Term Care Censuses 2001-2009 & Regional database (2014); author's calculations and mapping

| S 9*: Scatterplots of morbidity effects and mortality effects on the CFLY trend at age 65+ by care level and sex (only counties with expansion or compression included), higher symbol size indicates a higher estimation precision/a lower uncertainty* | |  |
| --- | --- | --- |
| Men, any care level   | Men, severe care level   | |
| Women, any care level   | Women, severe care level   | |
|  | | |

Note: counties are weighted by $1/\sum_{i=2001/03}^{2007/09} \left( \text{σ² (DFLY}_{i}) \right)$

Source: Statistical Offices of the Federation and the Länder, Statutory Long-Term Care Censuses 2001-2009 & Regional database (2014); author’s calculation

| S 10*: Scatterplots of morbidity effects and mortality effects on the CLY trend at age 65+ by care level and sex (only counties with expansion or compression included), higher symbol size indicates a higher estimation precision/a lower uncertainty* | |  |
| --- | --- | --- |
| Men, any care level   | Men, severe care level   | |
| Women, any care level   | Women, severe care level   | |
|  | | |

Source: Statistical Offices of the Federation and the Länder, Statutory Long-Term Care Censuses 2001-2009 & Regional database (2014); author’s calculation

| S 11*: Scatterplots of mortality effects on CFLY trend and mortality effects on CLY trend at age 65+ by care level and sex (only counties with expansion or compression included), higher symbol size indicates a higher estimation precision/a lower uncertainty* | |
| --- | --- |
| Men, any care level   | Men, severe care level   |
| Women, any care level   | Women, severe care level   |
|  | |

Source: Statistical Offices of the Federation and the Länder, Statutory Long-Term Care Censuses 2001-2009 & Regional database (2014); author's calculations
